# The Method and Study of Detecting Phenanthrene in Seawater Based on a Carbon Nanotube–Chitosan Oligosaccharide Modified Electrode Immunosensor

**DOI:** 10.3390/molecules28155701

**Published:** 2023-07-27

**Authors:** Yuxuan Wu, Wei Qu, Chengjun Qiu, Kaixuan Chen, Yuan Zhuang, Zexi Zeng, Yirou Yan, Yang Gu, Wei Tao, Jiaqi Gao, Ke Li

**Affiliations:** 1 College of Mechanical, Naval Architecture & Ocean Engineering, Beibu Gulf University, Qinzhou 535011, China; wyshwyx@163.com (Y.W.); qiuchengjun@bbgu.edu.cn (C.Q.); chenkx93@163.com (K.C.); zhuangyuan@bbgu.edu.cn (Y.Z.); zexi1998@163.com (Z.Z.); yanyirou0063@163.com (Y.Y.); a1059500787@163.com (Y.G.); 13332493888@163.com (W.T.); znkjgl@sina.cn (J.G.); like000304@163.com (K.L.); 2 Eastern Michigan Associated Engineering College, Beibu Gulf University, Qinzhou 535011, China; 3 College of Electronics and Information Engineering, Beibu Gulf University, Qinzhou 535011, China; 4 Guangxi Key Laboratory of Ocean Engineering Equipment and Technology, Qinzhou 535011, China

**Keywords:** multi-walled carbon nanotubes, chitosan oligosaccharide, phenanthrene, electrochemical immunosensor

## Abstract

Phenanthrene (PHE), as a structurally simple, tricyclic, polycyclic aromatic hydrocarbon (PAHs), is widely present in marine environments and organisms, with serious ecological and health impacts. It is crucial to study fast and simple high-sensitivity detection methods for phenanthrene in seawater for the environment and the human body. In this paper, a immunosensor was prepared by using a multi-wall carbon nanotube (MWCNTs)-chitosan oligosaccharide (COS) nanocomposite membrane loaded with phenanthrene antibody. The principle was based on the antibody–antigen reaction in the immune reaction, using the strong electron transfer ability of multi-walled carbon nanotubes, coupled with chitosan oligosaccharides with an excellent film formation and biocompatibility, to amplify the detection signal. The content of the phenanthrene in seawater was studied via differential pulse voltammetry (DPV) using a potassium ferricyanide system as a redox probe. The antibody concentration, pH value, and probe concentration were optimized. Under the optimal experimental conditions, the response peak current of the phenanthrene was inversely proportional to the concentration of phenanthrene, in the range from 0.5 ng·mL^−1^ to 80 ng·mL^−1^, and the detection limit was 0.30 ng·mL^−1^. The immune sensor was successfully applied to the detection of phenanthrene in marine water, with a recovery rate of 96.1~101.5%, and provided a stable, sensitive, and accurate method for the real-time monitoring of marine environments.

## 1. Introduction

Economic development and social progress have improved the quality of people’s lives, and have also led to increasingly serious environmental problems. Since various organic pollutants in the marine system cause harm to the marine environment and human health, the importance of monitoring and controlling the different types of organic pollutants in the marine environment has been put on the agenda [1]. Polycyclic aromatic hydrocarbons (PAHs) are organic compounds consisting of two or more monoaromatic rings or fused aromatic rings. Their main sources are transportation leakage, incomplete combustion, and the emissions of organic substances such as coal [2] and petroleum [3]. They are widely present in the environment through air and water [4], which not only pollute the environment, but also have a strong toxicity, carcinogenicity, and mutagenicity to organisms, especially the human body [5]. PAHs have different properties due to their different positions of aromatic ring composition. Phenanthrene (PHE) is the simplest tricyclic polycyclic aromatic hydrocarbon, and its composition includes a bay area. According to the “Bay Region Theory”, a spatially hindered region is formed between c-4 and c-5 of the phenanthrene structure [6], which makes it easy for phenanthrene to enter an organism and participate in its metabolism. It is easy for phenanthrene to form bay area diol epoxide and combine with DNA, RNA, and protein, resulting in genotoxicity, gene mutation, and carcinogenesis [7,8], and it has an impact on the heart [9], blood [10], and reproductive system [11] of organisms. Considering the impact of phenanthrene on the environment and biology, the World Health Organization listed phenanthrene as a category 3 carcinogen in 2017. Therefore, developing a fast, sensitive, and environmentally friendly PHE detection method has important practical significance for protecting human health and the ecological environment.

At present, the main methods for detecting trace levels of PAHs are fluorescence spectroscopy [12], surface-enhanced Raman scattering [13], liquid chromatography [14], chromatography-mass spectrometry [15], and the enzyme-linked immunosorbent method [16]. For the mainly used liquid chromatography and chromatography-mass spectrometry methods, although they have a high sensitivity and wide applications, they have many shortcomings, such as a high cost, complicated pre-processing, and long detection time. For ELISA and fluorescence spectroscopy, the detection is susceptible to light interference, which leads to inaccurate detection. Electrochemical immunoassays combine electrochemical analyses with immunoassays, immobilizing the antibodies on the electrode surface in advance and analyzing the signal changes before and after the reaction via the electrochemical method for qualitative or quantitative detection. It is widely used in various fields due to its advantages such as a high specificity, sensitivity, high stability, low cost, and easy automation [17,18,19].

Carbon nanotubes (MWCNTs) are composed of multi-layer graphene winding layers. Due to their tubular structure, carbon nanotubes have an excellent conductivity, high specific surface area, and biocompatibility [20,21]. They are an excellent modification material for making biosensors [22]. Functionalized carbon nanotubes can apply multi-walled carbon nanotubes to more fields. Carboxylated carbon nanotubes can provide carboxyl groups after introducing an acidic substance as a catalyst, such as sulphuric and nitric acids, and this carboxyl group enhances the condensation reaction with antibodies that are rich in hydroxyl groups to strengthen the antibody fixation, and also enable carbon nanotubes to play their role in more fields as nanomaterials [23].

Chitosan oligosaccharide (COS), a natural polysaccharide extracted from chitin, has a surface rich in a large number of amino and hydroxyl groups and exhibits a wide range of biomedical activities and film-forming properties, as well as electrocatalytic properties [24,25]. Chitosan oligosaccharides have properties similar to those of chitosan [26,27]. The addition of chitosan oligosaccharide can keep the antibody active and fix it more firmly, immobilizing it on the electrode surface. Its excellent medical activity also greatly reduces the background current in the detection system [28].

In this paper, a polycyclic aromatic hydrocarbon—phenanthrene—was used as the research object, the electrode was modified by carboxylated multi-walled carbon nanotubes—chitosan oligosaccharide nanocomposite membranes—and phenanthrene antibody was fixed on the surface of the nanocomposite membrane. An electrochemical immunosensor for the determination of phenanthrene in seawater was constructed based on cyclic voltammetry (CV) and differential pulse voltammetry (DPV). The electrochemical characteristics and detection capability, as well as the influencing factors of this sensor, were explored in detail.

In this study, a carbon nanotube–chitosan oligosaccharide composite combined with phenanthrene antibody was used to eliminate the interference of various substances in the complex system of seawater through the specific antibody–antigen recognition in the immune reaction, and to achieve the accurate capture of PAHs, phenanthrene, in the complex system of seawater. The electrochemical method combined with an immunosensor enabled the detection process to avoid complicated pre-treatment, shortened the detection time and detection cost, and was successfully applied to the detection of actual seawater samples.

## 2. Results and Discussion

### 2.1. Electrochemical Characterization of Immunosensors

#### 2.1.1. Morphological Features of the Sensor Platform

**Figure 1:**
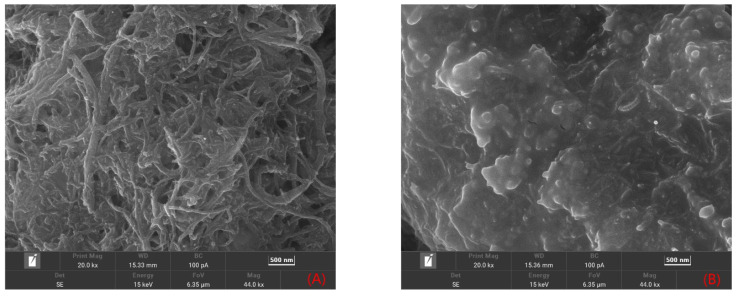

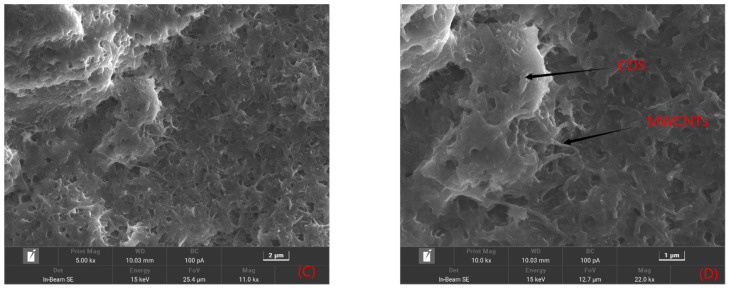
SEM images of carbon nanotubes (**A**), chitosan oligosaccharides (**B**), MWCNTs-COS (**C**) with 5 kx magnification, and MWCNTs-COS, and (**D**) composites with 20 kx magnification.

As shown in Figure 1A, MWCNTs have a tubular structure, and the electrostatic adsorption force made several MWCNTs interweave together to form a loose surface, which could significantly increase the reaction area. Figure 1B shows that, although the chitosan oligosaccharide molecule was large, it completely covered the electrode surface and formed a uniformly distributed film on the electrode surface. SEM electron micrographs of the carbon nanotube–chitosan oligosaccharide nanocomposite films are shown in Figure 1C,D. Figure 1C shows that the composite film at 5 kx magnification had a wrinkled shape on the electrode surface, and the chitosan oligosaccharides uniformly dispersed in the carbon nanotubes to form a loose network structure, which provided a larger specific surface area, as well as conductive channels to facilitate electron transfer. In Figure 1D, further enlarged SEM images show clear details; the MWCNTs wrapped around the chitosan oligosaccharide molecules, which was due to the abundant -NH_2_ and -OH of the chitosan oligosaccharide, forming noncovalent bonds with the MWCNTs, reducing the strong attraction between the MWCNTs, preventing MWCNT agglomeration, and increasing the stability of the composites.

#### 2.1.2. Performance Characterization of Electrochemical Immunosensors

**Figure 2:**
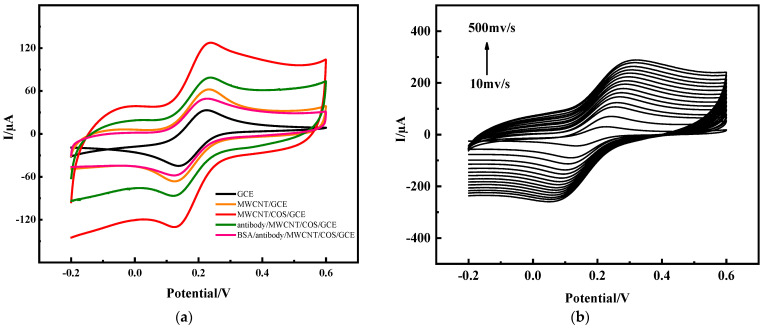
Construction process of electrochemical immunosensor in PBS (pH 7.0) buffer (**a**); CV (**b**) of immunosensor in PBS (pH 7.0) containing 5 mmol·L^−1^ [Fe(CN)_6_]^3−/4−^.

The immunosensor was placed in an electrolyte, which contained a 0.1 mmol·L^−1^ KCl and 5 mmol·L^−1^ K_3_Fe(CN)_6_ redox couple in PBS (pH = 7.0) buffer solution. The electrolyte was used as the redox reaction probe in the electrochemical reaction system, and the CV method and electrochemical impedance spectroscopy (EIS) were used to characterize the different properties of the different modified materials on the electrode surface. The cyclic voltammetry curve of the electrochemical immunosensor prepared in this paper is shown in Figure 2a. The CV test of the bare electrode (GCE) initially found a pair of stable and symmetrical redox peak currents, with a peak potential difference of about 88 mV and a peak current I_P_ of about 52.4 μA, which shows that the prepared test base had good electron transfer characteristics. The current peak increase in the electrodes modified by the carbon nanotubes was significant at 70.7 μA, which proved that the carbon nanotubes had a good conductivity and electron transfer ability. After coating the carbon nanotubes with another layer of chitosan oligosaccharide, the peak current increased to 111.7 μA. This was due to the protonation reaction of the basic chitosan oligosaccharide with the acidic acetic acid solution, and the accelerated electron transfer in the [Fe(CN)_6_]^3−/4−^ system. After fixing anti-PHE on the surface of the MWCNTs-COS nanocomposite membranes, it was found that the peak current decreased from 111.7 μA to 70.3 μA in the test base. This was due to the fact that the COS surface was rich in a large number of amino and carboxyl groups covalently bonded to the anti-PHE, and the large specific surface area of the carbon nanotubes and good biocompatibility made it easier to immobilize the antibodies, while the phenanthrene antibody (4D5) belonged to proteins, had insulation, and hindered electron transfer [29]. Therefore, the current was significantly reduced, which indicated that the nanocomposite membrane successfully immobilized the anti-PHE. After immobilizing the antibody, the electrode was placed in 3% BSA solution for 45 min to block the excess functional groups, thereby improving the specific recognition of phenanthrene and avoiding interference. Then, the electrodes were placed in the [Fe(CN)_6_]^3−/4−^ system for detection, and the current was further reduced to 61.1 μA due to the BSA hindering electron transfer.

The anti-PHE/MWCNTs-COS/GCE was placed in the electrolyte for CV scanning at different scanning speeds, and it was found that as the scanning rate increased, the peak current also increased, and within the range of 0.01~0.5 V·s^−1^, the current was linearly related to the square root of the scanning rate. It was proved that the process of electron transfer on the electrode surface in the redox system reaction was controlled by the diffusion process.

According to the Randles–Sevcik equation:

(1) $$I_{p}=2.69\times 10^{5}AD^{1/2}n^{3/2}v^{1/2}c$$

From this equation, the effective surface area of the electrode during the reaction can be calculated [30], where 

$I_{p}$ is the peak current; 

*A* is the effective surface area of the electrode (cm^2^);

*D* is the diffusion coefficient (cm^2^·s^−1^);

*n* is the number of moles of transferred electrons in the reaction system;

*v* is the potential change rate (0.05 V·s^−1^);

*c* is the concentration of ferrocyanide.

If *D* = 0.72 × 10^−5^ cm^2^·s^−1^ and *n = 1*, *c =* 5 mmol·L^−1^, the effective areas of GCE, MWCNTs/GCE, and MWCNTs-COS/GCE were calculated to be 0.065, 0.088, and 0.1386 cm^2^, respectively. It can be concluded that the synergistic effect of the two nanomaterials, the MWCNTs and chitosan oligosaccharide, significantly increased the area of the electrode surface involved in the reaction and improved the sensitivity of the electrochemical detection method.

**Figure 3:**
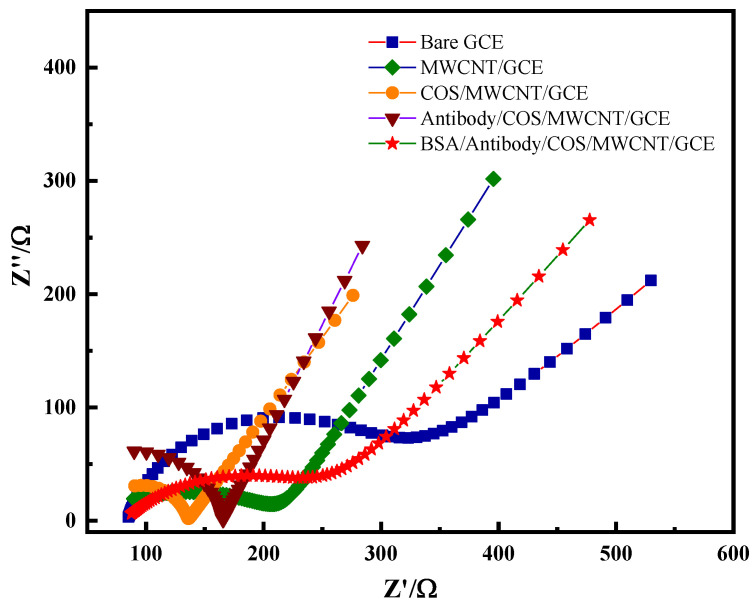
EIS diagram of immunosensor in pH 7.0 PBS solution.

Electrochemical impedance spectroscopy is a method of analyzing changes in the microscopic dynamics and diffusion of materials on an electrode or the surface of an electrode by measuring the change in impedance with the frequency of a sine wave. The electrochemical impedance spectrum is mainly divided into two parts: the semi-circle part, representing the high-frequency region, which is controlled by the electrode reaction force (charge transfer process), and the larger the semi-circle radius, the more difficult the electron transfer is in the electrochemical system, that is, the greater the resistance; and the linear part, representing the low-frequency region, which is controlled by the diffusion process of the reactants or products reacted by the electrode. As shown in Figure 3, compared with the impedance curve of the bare electrode, the impedance curve after modifying the carbon nanotubes on the electrode surface shows that the diameter of the semicircle was significantly reduced, indicating that the carbon nanotubes had a good conductivity and could accelerate the electron transfer rate in the system. After modifying the carbon nanotubes and chitosan oligosaccharide nanocomposite membranes, the semicircle radius was further reduced, which was due to the combination of protonated chitosan oligosaccharides and carbon nanotubes, promoting the diffusion of the potassium ferricyanide system on the electrode surface, thereby further accelerating the electron transfer rate. After further modification of the PHE antibody and BSA on the electrode surface, the radius of the semicircle gradually increased, indicating that the PHE antibody was successfully fixed on the surface of the carbon nanotube–chitosan oligosaccharide nanocomposite membrane. The reason for this was because PHE antibodies and BSA are proteins and their insulating properties reduce the rate of electron transfer on an electrode surface, that is, increase the resistance. The curve results for the CV curve and AC impedance spectrum were roughly the same, indicating that the carbon nanotube–chitosan oligosaccharide nanocomposite membrane not only had a good electron transfer ability, but also had an excellent signal amplification ability and was biocompatible, maintaining the activity of the antibody, so that the PHE antibody was successfully fixed on the electrode surface, which provides a good basis for subsequent work.

### 2.2. Optimization of Experimental Conditions

#### 2.2.1. Effect of Antibody Concentration on Immune Sensors

**Figure 4:**
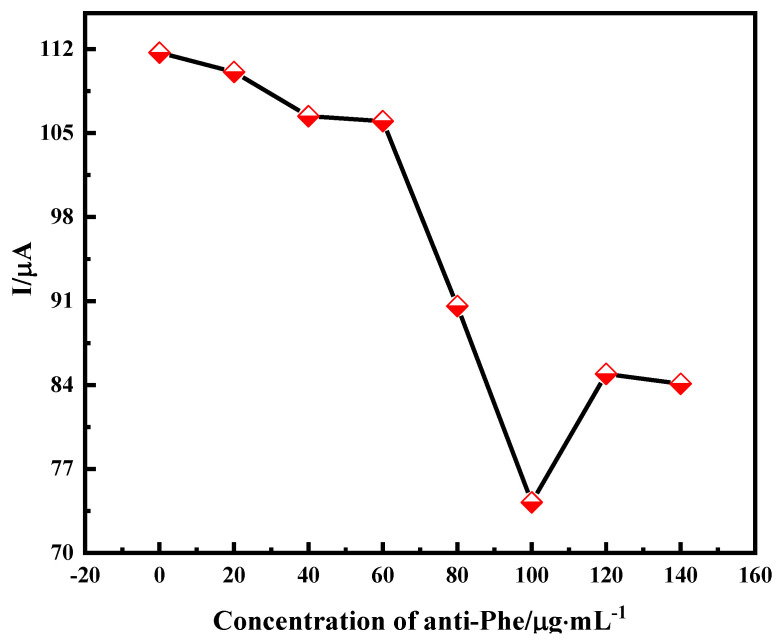
Effect of antibody concentration on antibody fixation amount of immune sensor.

In immunosensors, the amount of antibody fixed on the electrode surface largely determines the performance of the immunosensor. In this experiment, we incubated 10 μL of PHE antibody (4D5) solution at different concentrations in the same experimental environment. The PHE antibody was dropwise applied to the surface of the carbon nanotube–chitosan oligosaccharide nanocomposite membrane and scanned using DPV. As shown in Figure 4, the antibody concentration increased from 0 μg·mL^−1^ to 140 μg·mL^−1^, the peak current gradually decreased and reached a minimum value of 100 μg·mL^−1^, and the peak current increased when the antibody concentration continued to increase, which indicates that the antibody concentration on the electrode surface reached saturation due to the limited surface active sites of the carbon nanotube–chitosan oligosaccharide nanocomposite membrane. Therefore, 100 μg·mL^−1^ was selected as the optimal antibody concentration for the experiment.

#### 2.2.2. Effect of pH on Immune Sensors

**Figure 5:**
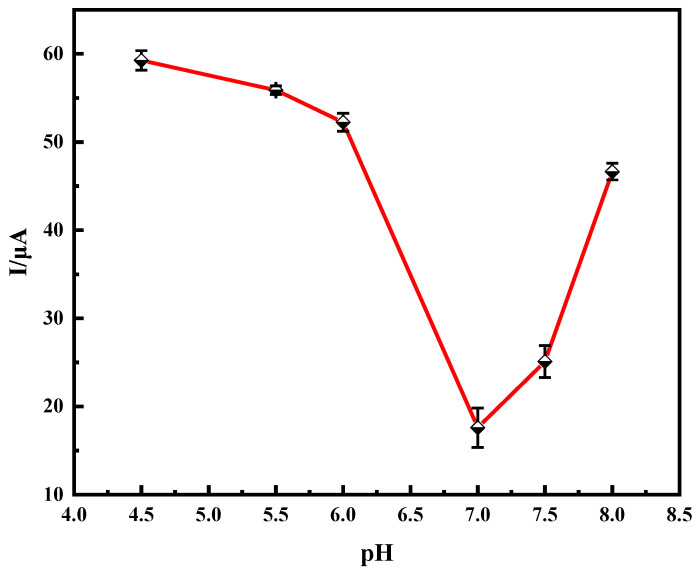
pH optimization experiment.

Among the factors influencing immune sensors, pH is an important factor. The pH value of the buffer in the redox system had a significant effect on the activity of the PHE antibodies and total amount of antibodies fixed on the electrode surface. Buffer solutions with different pH values were prepared, and their corresponding response currents were measured using DPV. As shown in Figure 5, in the 4.5 to 7.0 range, the response current decreased with an increasing pH and reached a minimum value at 7.0; in the 7.0 to 8.0 segment, it increased with an increasing pH. The obtained relative standard deviation (RSD) value was between 0.873~2.025% (*n* = 3). The experiments showed that the prepared immune sensor worked best at pH 7.0, because the antibody reduced its activity under acidic or alkaline conditions. Thus, a buffer solution with a pH of 7.0 was chosen.

#### 2.2.3. Probe Concentration on the Immunosensor

In this experiment, a potassium ferricyanide system was used as the probe for the redox reaction, and a change in the potassium ferricyanide concentration would directly affect the detection current, so it was necessary to optimize the probe concentration. Potassium ferricyanide solutions (1, 5, 10, and 15 mmol·L^−1^) were prepared as the electrolyte, the peak current change before and after the immune reaction was detected using the CV method, the data setting of the CV method was −0.2~0.6 V, and the sweep speed was 0.05 V·s^−1^. The oxidation current value and reduction current value of the immunosensor in different concentrations of the test base solution before the reaction were recorded, the immunosensor was placed in PHE solution and incubated at 37 °C for 30 min, and immediately after being taken out, it was detected and recorded in different concentrations of the test base. It can be seen from the change in the redox current before and after the reaction in Table 1, when the probe concentration was 5 mmol·L^−1^, the current changed the most. Therefore, a probe concentration of 5 mmol·L^−1^ was selected.

### 2.3. Performance of Immunosensors

#### 2.3.1. Detection of PHE Using Differential Pulsed Voltammetry (DPV)

**Figure 6:**
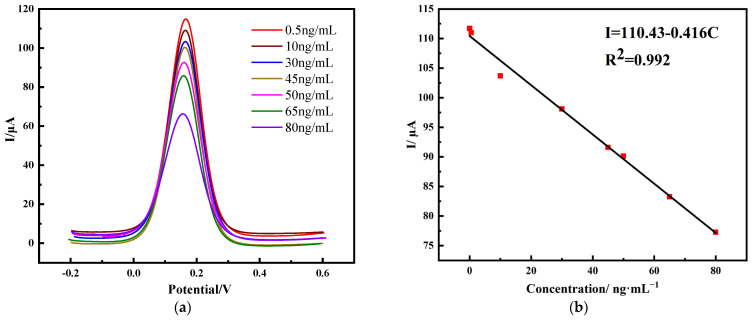
DPV response of immunosensors at different concentrations of PHE (0.5, 10, 30, 45, 50, 65, 75, and 80 ng·mL^−1^) The electrolyte buffer contains 0.1 mmol·L^−1^ KCl + 5 mmol·L^−1^ K_3_Fe(CN)_6_ in 25 mL PBS (pH 7.0) (**a**). Calibration curves for PHE immunosensors at different concentrations (**b**).

For immune sensors, their performance indicators include many aspects, the most important of which are the effective response range of the sensor and its response sensitivity. Under the optimal experimental conditions, after multiple sets of experiments, the response current signal values corresponding to the different concentrations of PHE were detected using the DPV method. The potential waveform of DPV was formed by the superposition of the step-wave reference potential and short-time potential pulse, which made the output current signal of DPV the difference in the current subtraction, so that the DPV obtained a higher sensitivity and lower detection limit than the CV. The principle was that the electrode surface antibody–antigen reaction led to changes in the electron transfer, and the electrode surface induced a current to reflect the content of the PHE. Therefore, when using DPV detection, the more phenanthrene content in the sample, the lower the peak current. However, since PHE is a polycyclic aromatic hydrocarbon and is easily electrooxidized to promote electron transfer at a high potential, this can make the detection current larger to obtain a wider detection range. Thus, the oxidation peak of DPV was selected for a quantitative analysis of the PHE content. As shown in Figure 6, the detected electrode surface response current was inversely proportional to the concentration of PHE, that is, the peak current value decreased with an increase in the PHE concentration. According to the linear equation I = 110.43 − 0.416C, the detected response current was linearly correlated with the concentration of PHE in the sample, from 0.5 ng·mL^−1^ to 80 ng·mL^−1^, and the correlation coefficient R^2^ was 0.992. The slope parameter of the calibration equation was used to calculate the detection limit, according to the signal-to-noise ratio (SNR) = 3, the blank sample was tested in parallel for seven times, and the detection limit calculation formula was obtained to obtain a detection limit of 0.30 ng·mL^−1^, which was lower than the international standard. We compared and summarized the detection method in this paper with previous methods, which are reflected in Table 2. The MWCNTs/COS/anti-PHE/GCE prepared in this paper had the advantages of simple preparation, rapid detection, and a low detection limit. Due to the excellent physicochemical properties of nanomaterials, immunosensors have a wide detection range and low detection limits.

#### 2.3.2. Specificity, Reproducibility and Stability of Immune Sensors

**Figure 7:**
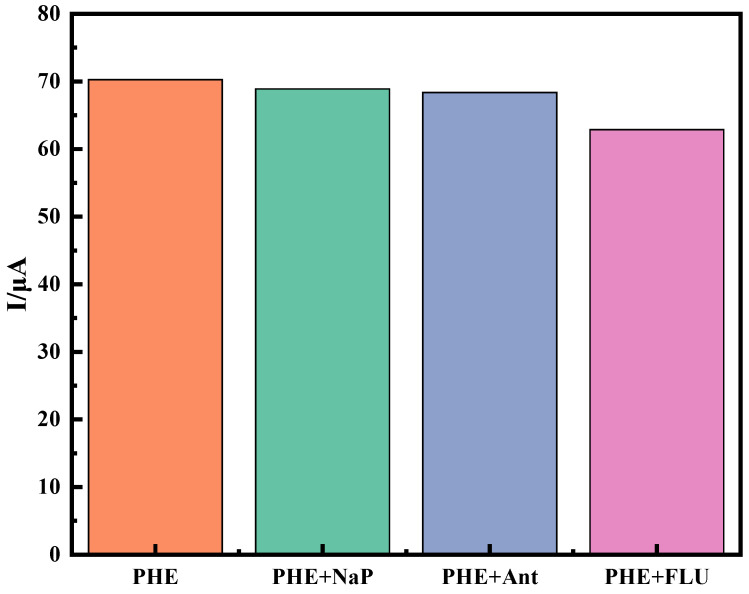
Immunosensor-specific experiment.

Specificity, reproducibility, and stability are the key parameters for judging the performance of immune sensors in practical applications. The specificity of an immune sensor determines whether it can accurately identify the target substance in complex components. Under the optimal experimental conditions, naphthalene (Nap), anthracene (Ant), and fluoranthene (Flu) were selected as interfering substances. In Figure 7, After 30 min of incubation of the immunosensor in a 50 ng·mL^−1^ PHE standard solution, the response signal was detected using DPV. Equal amounts of naphthalene, anthracene, and fluoranthene were added to the PHE standard solution. Under the same experimental conditions, the response current value of DPV was determined separately. Compared to the PHE standard solution, the peak current decreased by 1.96%, 2.0%, and 2.71%, respectively, and the current change was within 3%. The test results showed that, under the optimal experimental conditions, the interferers did not significantly interfere with the immune sensor of this experiment.

**Figure 8:**
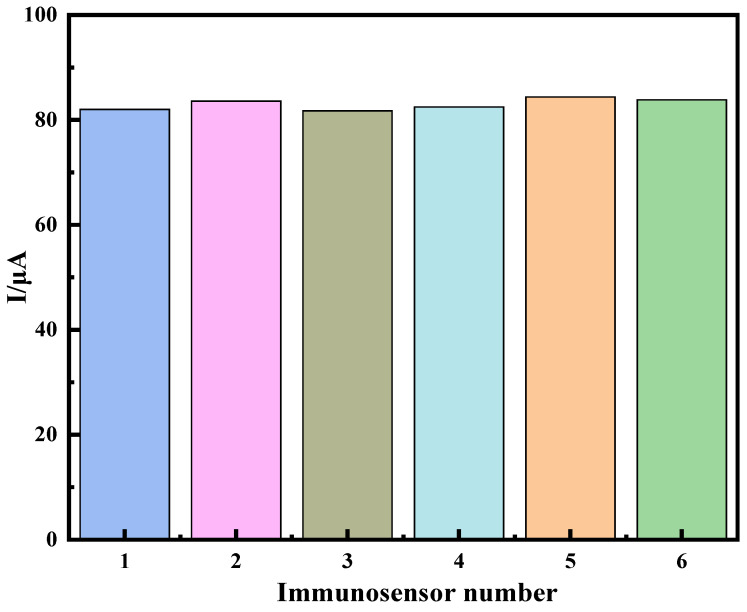
Immunosensor reproducibility experiment.

Reproducibility can assess whether an electrochemical immunosensor is accurate. In the experiment, six identical modified electrodes were prepared to detect 50 ng·mL^−1^ of PHE standard solution three times under the same conditions, and the relative standard deviation (RSD) of the detection results was 1.289%, as shown in Figure 8. Six modified electrodes detected a PHE of 49.8 ng·mL^−1^ under the same conditions, with an RSD of 0.283%. The detection results showed that the electrochemical immunosensor had a good reproducibility.

The prepared electrochemical immunosensor was placed in a 5 mmol·L^−1^ [Fe(CN)_6_]^3−/4−^ + 0.1 mol·L^−1^ KCl + 25 mL PBS test base and recorded using CV scanning for 20 consecutive cycles. The CV curves almost overlapped and the current drop was only 1.91%. The immune sensor coated with PHE antibody was sealed and stored at 4 °C, a DPV scan was performed again 14 days later, and the scanning results showed that the peak current dropped to 97.15% of the original value, without a significant change. The results show that the bare electrode was electrochemically stable after modification with carbon nanotubes and chitosan oligosaccharides, which could fix the antibody more firmly and maintain the antibody activity, which gave the immune sensor a good stability.

### 2.4. Actual Sample Determination

The prepared immunosensor was applied to the actual water sample test and a spike recovery experiment was used to investigate its accuracy. The water samples were taken from the coastal surface seawater in the Fangcheng Port area, filtered using a 0.22 μm filter membrane, and their pH was adjusted to 7.0 by NaOH. Differential pulsed voltammetry (DPV) was used to detect seawater samples, and the content of coastal surface seawater in Fangcheng Port Sea area was 97.31 ng·mL^−1^. Then, phenanthrene standard samples (10, 20, 30, and 40 ng·mL^−1^) were added for the spike recovery experiments, and the results are shown in Table 3. It can be seen from Table 3 that the recovery rate was 96.1~101.5%, indicating that the accuracy of the sensor was good.

## 3. Materials and Methods

### 3.1. Chemicals

MWCNTs (purity > 98% wt, < 8 nm outer diameter, and length 10–30 μm) were purchased from Chengdu Organic Chemistry Company, Ltd., Chengdu, China, Chinese Academy of Sciences, and chitosan oligosaccharide (COS), MW < 3000, and PBS buffer were purchased from Shanghai Yuanye Biotech Co., Shanghai, China, Bovine serum protein (BSA), phenanthrene (PHE), acetonitrile (AR), and potassium ferricyanide (K_3_Fe(CN)_6_) were purchased from Shanghai Macklin Biochemical Technology Company, Ltd., Shanghai, China, acetic acid (GR) and sodium hydroxide (AR) were purchased from Sinopharm Chemical Reagent Company, Ltd., Shanghai, China, and 1-ethyl-(3-dimethylaminopropyl) carbodiimide hydrochloride (EDC), N-hydroxysuccinimide (NHS), and phenanthrene antibody (4D5), sc-69886 were purchased from Santa Cruz, Shanghai, China. 4D5 is a mouse monoclonal clone IgG_2b_.

### 3.2. Apparatus

The apparatus used were a CHI660E electrochemical analyzer (Shanghai Chenhua Instrument Company, Shanghai, China); magnetic stirrer, electronic analytical balance, ultrasonic cleaner, and high-speed centrifuge (Shanghai lichen Instrument Technology Co., Ltd.); electric thermal blast dryer (Shanghai Shangyi Instrument Equipment Co., Ltd., Shanghai, China), laboratory pure water, and ultrapure water system (Nanjing EPED Science and Technology Development Co., Ltd., Nanjing, China)

### 3.3. Experimental Processes

#### 3.3.1. Preparation of Multi-Walled Carbon Nanotube–Chitosan Oligosaccharide Composite Membranes

First, 100 mg of MWCNTs was precisely weighed using an electronic analytical balance and added to 200 mL of nitric acid (HNO_3_, 2.0 mol·L^−1^), then cooled to room temperature, and rinsed with deionized water. Then, this was added to 120 mL of concentrated HNO_3_ and a concentrated sulfuric acid (H_2_SO_4_) mixed acid solution (the volume of HNO_3_ and H_2_SO_4_ was 1:3), and sonicated for 3 h. It was then cooled to room temperature and filtered using a film with a pore size of 0.45 μm. Next, the products were washed repeatedly with deionized water to neutrality. Finally, the extracted and filtered products were dried in a drying oven at 120 °C for 1.5 h to remove the excess moisture from the MWCNTs. The prepared carboxylated MWCNTs were placed in a beaker and sealed for storage. In total, 0.01 g of carboxylated MWNTs was dissolved in 10ml of PBS (pH = 7.0) buffer, centrifuged in a centrifuge at 15,000 r/min for 10 min, sonicated in an ultrasonic machine for 1 h, and stirred at room temperature for 1 h. A total of 0.02 g of chitosan oligosaccharide was dispersed in dilute acetic acid (1% *v/v*) to prepare a solution of 0.02 ng·mL^−1^ of chitosan oligosaccharide acetic acid and stored at 4 °C. Chitosan oligosaccharides are rich in amino groups and hydroxyl groups and form a stable non-covalent bonds with carboxyl groups on the surface of carbon nanotubes. The nano-scale composite films formed by the two groups can not only promote electron transfer, but also provide a platform for antibody immobilization. The GCE was polished to the mirror surface on a polishing cloth with 0.5 μm, 0.1 μm, and 0.05 μm alumina polishing powder, then rinsed with dilute nitric acid, absolute ethanol, and ultra-pure water, and sonicated for 30 s. After pretreatment, the GCE was placed in a 0.5 mol/L H_2_SO_4_ solution, cyclic voltammetry (CV) was used, and the potential parameters were −0.3 V to +1.5 V for the activation scanning, which was performed 10 times to activate the electrode. Then, this was rinsed with ultrapure water and dried with nitrogen. The CV method (−0.2 V~0.6 V) was used in an electrolyte, which contained 0.1 mmol·L^−1^ KCl and 5 mmol·L^−1^ K_3_Fe(CN)_6_ in PBS (pH = 7.0) buffer solution until the oxidation–reduction peak was symmetrically stable and the potential difference was about 90 mV. A total of 5.0 μL of carboxylated carbon nanotubes were vertically dropped onto the electrode surface and placed in a desiccator to dry for 10 min. A further 10 μL of EDC/NHS (1:1) mixture was added drop by drop and placed at 4 °C for 2 h to activate the carboxyl group. Using a pipetting gun, 5 μL of COS solution was dropped onto the electrode surface and dried in air. 

#### 3.3.2. Preparation of Electrochemical Immunosensors

Firstly, PHE antibody (4D5) (10 μL) was dropped onto the surface of the MWCNTs-COS nanocomposite film and incubated in a desiccator for 2 h at 37 °C. Then, 5 μL of 3% BSA solution was added at 37 °C for 40 min to block the remaining active sites, which allowed for the specific recognition of phenanthrene by the immunosensor. Finally, the MWCNTs/COS/anti-PHE/BSA modified immunosensors were stored at 4 °C for later use.

#### 3.3.3. Immunoassay for Phenanthrene Detection

**Figure 9:**
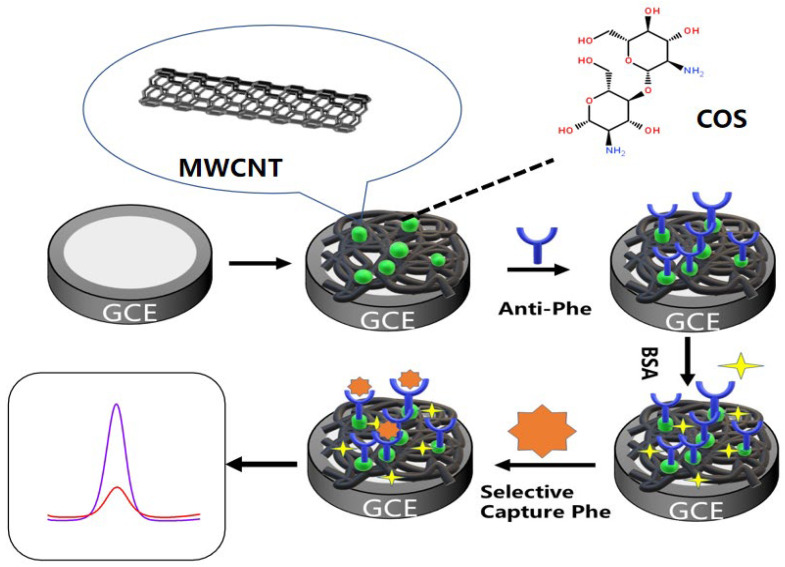
Schematic illustration of the detection mechanism of the electrochemical immunosensor. The purple line is a 0.5 ng·mL^−1^ content phenanthrene solution; The red line is 80 ng·mL^−1^ phenanthrene solution.

The immunosensor was placed in electrolyte containing with 0.1 mmol·L^−1^ KCl and 5 mmol·L^−1^ K_3_Fe(CN)_6_ in PBS (pH = 7.0) buffer solution. Cyclic voltammetry (CV) and electrochemical impedance spectroscopy (EIS) were used to characterize the modified materials and the immunosensor properties. The potential was set at −0.2 V to 0.6 V, and the scanning rate was 0.05 V·s^−1^. Before each measurement, 10 μL of phenanthrene standard solutions with different concentrations (0.5~80 ng·mL^−1^) were dropped onto the surface of the modified immunosensor and incubated at 37 °C for 30 min. Finally, the three electrodes (working electrode, counter electrode, and reference electrode) were immersed in the electrolyte, and DPV was used to measure the peak current I_P_ of the different concentrations of the phenanthrene. The DPV was set to be 50 mV in amplitude and −0.2 V to 0.6 V in potential. By analyzing the peak current data and the standard concentrations of the phenanthrene, a linear relationship was established to obtain a standard curve. All the electrochemical measurements were performed at room temperature. The modification process and detection mechanism are shown in Figure 9.

## 4. Conclusions

In this paper, MWCNTs-COS nanocomposite membranes were prepared, and an electrochemical immune sensor for PHE was constructed based on the composite membrane immobilization of PHE antibodies. The excellent conductivity and high specific surface area of carbon nanotubes were used to enhance the electron transfer rate in the detection process to improve the detection sensitivity. Chitosan oligosaccharides provided good film-forming and a large number of amino groups fixed the PHE antibodies more and more firmly on the electrode surface, strengthening the stability of the immune sensors. The optimal experimental conditions were determined: an antibody concentration of 100 μg·mL^−1^, a pH of 7.0, and a probe concentration of 5 mmol·L^−1^. After the optimization experiment, the linear response range of the PHE was 0.5~80 ng·mL^−1^, and the detection limit was 0.30 ng·mL^−1^.

Compared with the traditional method, the immunosensor eliminated the complex pretreatment process, had the advantages of a simple operation, rapid detection, high selectivity, and strong stability, etc., was fast and accurate in a seawater system, and was easy to operate. It has excellent application value for environmental monitoring.

## Figures and Tables

**Table 1:** Optimization of Probe Content.

k_3_ Fe(CN)_6_	Ipa	I’pa	ΔIpa	Ipc	I’pc	ΔIpc
c/(mmol·L^−1^)	(μA)	(μA)	(%)	(μA)	(μA)	(%)
1	−24.4	−23.99	1.68%	17.76	17.59	0.95%
5	−57.75	−46.17	20.05%	47.19	34.54	26.80%
10	−125.4	−118.5	5.50%	100.1	97.96	2.14%
15	−289.9	−275.0	5.13%	295.1	293.8	0.44%

**Table 2:** Linear ranges and detection limits of different ways for PHE.

Method	Linear Range (ng·mL^−1^)	LOD (ng·mL^−1^)	Reference
MNPs-Phe-McAb/CLEIA	1.85–71.51	0.85	[31]
Cyclodextrin/Au NPs/SERS	-	0.1–0.9	[32]
aluminum film/ultraviolet (UV) fluorescence	0–250	0.49	[33]
MWCNTs/COS/anti-PHE/GCE	0.5–80	0.30	this work

**Table 3:** Electrochemical immunosensor recovery test.

Initial (ng·mL^−1^)	Add (ng·mL^−1^)	Found (ng·mL^−1^)	Recovery (%)
97.31	10	106.92	96.1
-	20	116.95	98.2
-	30	127.04	99.1
-	40	137.93	101.5

## Data Availability

Not applicable.
